# Utilizing Stable Gene‐Edited Knockout Pools for Genetic Screening and Engineering in Chinese Hamster Ovary Cells

**DOI:** 10.1002/biot.70033

**Published:** 2025-05-16

**Authors:** Jannis Peter Marzluf, Kirchmeier Daniela, Jennifer Klein, Christoph Zehe, Ann‐Cathrin Leroux

**Affiliations:** ^1^ Department of Gene Therapy University of Ulm Ulm Germany; ^2^ Sartorius Stedim Cellca GmbH Ulm Germany

**Keywords:** CHO, clonal heterogeneity, genetic engineering, host cell line engineering, stable CHO pools

## Abstract

Chinese hamster ovary (CHO) cells are the primary host for biopharmaceutical production. To meet increasing demands for productivity, quality, and complex molecule expression, genetic engineering, particularly clustered regularly interspaced short palindromic repeats (CRISPR)‐mediated gene knockout (KO), is widely used to optimize host cell performance. However, systematic screening of KO targets remains challenging due to the labor‐intensive process of generating and evaluating individual clones. In this study, we present a robust, high‐throughput CRISPR workflow using stable KO pools in CHO cells. These pools maintain genetic stability for over 6 weeks, including in multiplexed configurations targeting up to seven genes simultaneously. Compared to clonal approaches, KO pools reduce variability caused by clonal heterogeneity and better reflect the host cell population phenotype. We demonstrate the utility of this approach by reproducing the beneficial phenotypic effects of fibronectin 1 (FN1) KO, specifically prolonged culture duration and improved late‐stage viability in fed‐batch processes. This workflow enables efficient identification and evaluation of promising KO targets without the need to generate and test large numbers of clones. Overall, screening throughput is increased 2.5‐fold and timelines are compressed from 9 to 5 weeks. This provides a scalable, efficient alternative to traditional clonal screening, accelerating discovery for CHO cell line engineering for biopharmaceutical development.

## Graphical Abstract and Lay Summary

A pooled CRISPR knockout (KO) workflow enables high‐throughput genetic KO screening in CHO cells without the need for clonal isolation. KO pools remain genetically stable in both singleplex and multiplex formats and are able to reproduce phenotypic KO effects from isolated clones. This workflow accelerates screening by reducing timelines from 9 to 5 weeks and increasing throughput 2.5‐fold over traditional clonal KO screening approaches.

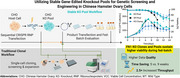

**Abbreviations**
Cas: CRISPR‐associated protein
CHO: Chinese hamster ovary
CRISPR: clustered regularly interspaced short palindromic repeats
DNA: desoxyribonucleic acid
FITC‐LCA: fluorescein isothiocyanate labeled lens culinaris agglutinin
FN1: fibronectin 1
FUT8: α1,6‐fucosyltransferase 8
HE: high efficiency
InDel: insertion/deletion
KO: knockout
LE: low efficiency
LPL: lipoprotein lipase
PCOLCE: procollagen C‐endopeptidase enhancer
PXDN1: peroxidasin 1
RNA: ribonucleic acid
RNP
: ribonucleoprotein
sgRNA: single guide RNA
THBS1: thrombospondin 1
VCC: viable cell concentration

## 1 Introduction

Despite significant advancements in the manufacturing of biopharmaceuticals using Chinese hamster ovary (CHO) cells over the past decades [1], there is a strong economic demand for strategies that increase bioprocess productivity, enhance stability of cell lines, and enable the engineering of specific desirable traits in CHO cells [2, 3, 4, 5, 6]. The discovery of clustered regularly interspaced short palindromic repeats (CRISPR)/CRISPR‐associated protein (Cas)9 systems as a gene‐editing tool in addition to analytical methods in transcriptomics, metabolomics, and proteomics have enabled unprecedented insight into the inner workings of production cell lines, making the engineering of superior host cell lines feasible. Genetic manipulations have been shown to effectively reduce apoptosis [2, 7], improve product stability [8, 9], and eliminate antibody fucosylation [10]. Further studies have shown the increased productivity and secretion capacity of CHO cells [11, 12, 13] and reduced host cell protein levels [14, 15] following genetic alterations. Although the generation of engineered host cell lines has become more prevalent, the labor‐intensive screening required to identify correctly edited single cell clones significantly limits the number of clones screened in studies investigating the effects of genetic modifications. This limitation is particularly pronounced in multiplexed studies where multiple gene edits are necessary. For instance, isolating single‐cell decaplex knockout (KO) clones required the screening of approximately 704 clones to ultimately identify just six successfully edited KO clones [16]. To generate multigene KO cells, one might reduce the numbers of genes addressed in a single transfection and add multiple steps of subcloning. To this end, some studies have shown an effective multiplexed approach to KO multiple genes in a single cell to reduce the host cell protein profile [15] by reducing the number of genes knocked out per round and performing sequential rounds of editing and cloning.

In addition, investigating a low number of single‐cell clones as a proxy to identify genetic engineering effects has notable limitations. Single‐cell cloning introduces significant heterogeneity within the derived population [17, 18, 19, 20, 21]. CHO cells exhibit an inherent aneuploid unstable genome with frequent genomic rearrangements, changes in single‐nucleotide polymorphisms, and significant copy number variations [22]. Additionally, during single cell cloning, individual cells may undergo substantial genomic rearrangements [20] and changes in desoxyribonucleic acid (DNA) methylation [17, 23], which in turn also affect clone characteristics. Many studies rely on a limited number of KO clones, often fewer than 10–15, sometimes even just single KO clones to screen for KO effects. These inherently highly variable clones are used for comparison against also highly variable wild‐type (WT) clones. This limited sample size increases the risk of biased results and challenges the reproducibility of findings, particularly due to the high clonal variability apparent in CHO cells. This high level of heterogeneity in single‐cell clones complicates the reliable assessment of KO‐induced phenotypic changes or the lack thereof. Moreover, performing sequential rounds of editing and cloning introduces an inherent selection bias, where the initial choice of clone exerts a founder effect that propagates through subsequent rounds of engineering and screening.

Nevertheless, most studies investigate an insufficient number of clones to accurately assess the effects of KOs on changes in cell bioprocess behavior. Here, we present a novel screening system to evaluate KO effects directly at the pool stage, achieving high KO efficiencies in heterogeneous KO pools through multiple transfections and demonstrating both genotypic and phenotypic stability of heterogeneous KO pools. Using this system, we confirmed the previously identified (unpublished) beneficial effects of a fibronectin 1 (FN1) KO as a genetic modification that enhances bioprocess duration, with final titers increased up to two‐fold and an improved bioprocess growth behavior in shake flask fed‐batch bioprocess.

## 2 Methods

### 2.1 Cell Culture

CHO DG44 cells were obtained from Lawrence Chasin (Columbia University, NY) and in‐house adapted to serum‐free suspension growth in chemically defined medium. The cells were cultured in serum‐ and antibiotics‐free CD DG44 media (Gibco, Carlsbad, CA, USA) supplemented with 6 mM glutamine (Sartorius Stedim Cellca GmbH, Ulm, Germany) and 0.001% Pluronic F‐68 (Gibco, Carlsbad, CA, USA). CHO cells were cultivated in 50 mL in 250 mL shake flasks (Corning, NY, USA) being passaged every 2 days to an inoculation density of 2E5 cells/mL. Following transfection, cells were cultivated in 1 mL in 24‐well plates (Nunc, Roskilde, Denmark) and after sufficient cell growth expanded to 4 mL in 6‐well (Nunc, Roskilde, Denmark) and then to 25 mL in 125 mL shake flasks (Corning, NY, USA) for cell banking and further experimentation. All cultures, except 24‐, 96‐, and 384‐well plates, were shaken at 110 rpm linear and cultivated in 80% humidity at 36.8°C and 7.5% pCO_2_ unless otherwise noted (standard conditions). Cell counting for routine cell cultivation and expansion was done using the CASY Cell Counter & Analyzer (OMNI Life Science, Bremen, Germany).

### 2.2 Synthetic gRNA Design and Screening

Gene targets for this study are shown in Table 1. Guide RNA sequences were designed using Geneious Prime software, version 11.0.11 (Biomatters, Auckland, New Zealand), or the CRISPR Guide RNA Design software (https://www.benchling.com) targeting an early exon present in all transcript variants to induce early frameshift mutations, rendering the translated protein unfunctional. gRNAs were selected based on scoring on‐target efficiency [24] and off‐target specificity [25]. It was ensured that possible off‐targets reside in nonprotein‐coding sequences of the host genome. Off‐target scoring was performed against a proprietary, fully sequenced, and annotated genome assembly of the host cell line (Sartorius Stedim Cellca GmbH). Three single guide RNAs (sgRNAs) were screened for each target gene to use the highest efficiency sgRNA in downstream experimentation. sgRNAs were ordered as TrueGuide Snythetic gRNAs at ThermoFisher Scientific (Thermo Fisher Scientific, Waltham, MA, USA) and transfected with TrueCut Cas9 Protein v2 (Thermo Fisher Scientific) in 1:1 ratio as 7.5 pmol preassembled ribonucleoprotein (RNP) into 2E5 cells using a NEON Transfection System and NEON Transfection System 10 µL Kit (Thermo Fisher Scientific). Transfection parameters were set to 1700 V, 20 ms pulse width, and 1 pulse. The RNP‐transfected host cells were subjected to genotyping by genomic DNA extraction, PCR, and Sanger‐sequencing analysis followed by Inference of CRISPR Edits (ICE)‐analysis 48 h after transfection as described in section 2.3.

**TABLE 1:** Gene target products used in this study.

Gene	Full name	Function/role	UniProt ID
FUT8	Fucosyltransferase 8	Involved in fucosylation, a key glycosylation process	G3HCE4
FN1	Fibronectin 1	ECM protein involved in cell adhesion and migration	A0A3L7IDB0
LPL	Lipoprotein lipase	Catalyzes hydrolysis of triglycerides	A0A3L7IKX6
NID1	Nidogen 1	Connects laminin and collagen in the ECM	A0A3L7HVW3
PCOLCE	Procollagen C‐endopeptidase enhancer	Modulates collagen processing and ECM remodeling	G3I664
PXDN	Peroxidasin	Involved in ECM formation and H_2_O_2_ signaling	A0A3L7HCC3
THBS1	Thrombospondin 1	Mediates cell‐to‐cell and cell‐to‐matrix interactions	G3HHV4
BGN	Biglycan	Small proteoglycan involved in ECM structure	A0A061HUR7

*Note*: Shown are gene symbol, full name, function, and UniProt ID.

### 2.3 Genotyping of Pools and Clones

Genomic DNA was extracted from transfected pools or single cell clones using the QuickExtract DNA Extraction Solution (Lucigen), followed by PCR to amplify the genomic regions flanking the sgRNA cut site aiming for 200–300 basepairs up‐ and downstream. Amplicons were sent for PCR purification and Sanger sequencing at Microsynth Seqlab GmbH (Göttingen, Lower Saxony, Germany). The Sanger sequencing traces for each test sample (edited) and its corresponding control sample (unedited) were uploaded to the ICE software tool, version 3.0 (ice.synthego.com) (Synthego, Menlo Park, USA) and analyzed according to the developer's instructions. ICE analysis provides the insertion/deletion (InDel) percentage and the KO score. The InDel percentage reflects the editing efficiency of the edited trace compared to the control trace, irrespective of whether the InDel results in a frameshift. The KO score indicates the KO efficiency, representing the proportion of cells with either a frameshift InDel or a fragment deletion, which is likely to result in a functional KO. The gRNA with the highest KO score for a given gene target was selected for high editing efficiency and a favorable InDel profile, characterized by predominantly out‐of‐frame InDels and a high KO score, with a preference for sgRNAs that produce a limited variety of InDels, ideally a single dominant type.

### 2.4 Generation of KO Clones and High KO Proportion Pools

To generate KO populations of the CHO DG44 host cell for all target genes, transfections were performed using the previously selected best sgRNA out of the three screened sgRNAs. These single RNP‐transfected pools were subsequently used for single‐cell cloning to derive KO clones for further experimentation. Single‐cell clones were generated using high‐throughput nanowell‐based image‐verified cloning with the CellCelector (Sartorius ALS GmbH, Jena, Germany). Each well of a 24‐nanowell plate was filled with proprietary 0.5 mL cloning medium. The plate was centrifuged (800 x *g*, 5 min) to fully settle the media into the bottom grid of the nanowells ensuring full liquid contact. A total of 2000 cells were seeded into these wells by diluting the target cells to 4000 cells/mL in cloning medium and seeding 0.5 mL of this dilution into one nanowell. Ahead of seeding, the sample was passed through a cell strainer (Corning, NY, USA). Each of the 24‐wells contains approximately 4400 nanowells, resulting in <0.5 cells being seeded per well. At the end of seeding, the plate was again centrifuged (300 x *g*, 3 min) to settle all cells. Cells are scanned using the CellCelector (Sartorius ALS GmbH) on the day of seeding to ensure monoclonality and then again after 4 days to assess growth and pick outgrown clones (15–20 cells) into 384 well‐plate containing standard cultivation media. Confluency of clones was measured using the CellMetric (Solentim, Bournemouth, UK) intermittently from Days 7 to 14. Once most of the clones reached 70%–100% confluency, all clones were transferred to 96‐well plate and passaged every 3–4 days while genotyping took place. Full KO clones were expanded to 12‐well plates in 1.5 mL standard cultivation media before expansion to 6‐well and shake flask under standard conditions. For the generation of KO pools with high KO scores, the same RNP transfection was performed three sequential times 48 h apart, with the same cells. These pools were then expanded for further experimentation.

### 2.5 FITC‐LCA Assay for Functional Analysis of FUT8 KO Pools and Clones

To assess the phenotypic outcome of RNP transfections targeting the α1,6‐fucosyltransferase 8 (FUT8) gene, pools were stained with fluorescein isothiocyanate labeled lens culinaris agglutinin (FITC‐LCA), which binds to fucose residues on membrane proteins. LCA has also been previously used to selectively enrich FUT8 KO subpopulations [26]. A mixture of FITC‐positive and FITC‐negative cells is expected in CRISPR‐edited pools, with FUT8 KO cells showing a complete shift to FITC‐negative populations. This assay was employed to validate the results from ICE analysis as an orthogonal method, enabling the measurement of phenotypic outcomes in addition to genetic outcomes. It was used to assess KO efficiency of different FUT8‐targeting sgRNAs at the pool level. Untransfected CHO DG44 host cells were used as a negative control. Cells were prepared by centrifugation (390 x *g*, 3 min) and washing twice with cold CASYton buffer (OMNI Life Science, Bremen, Germany). Staining was performed by incubating cells with 5 µg/µL FITC‐LCA (Thermo Fisher Scientific) for 30 min at 4°C, followed by two additional wash cycles. The fluorescence was measured by excitation at 488 nm using the IntelliCyte iQue Screener (Sartorius, Göttingen, Germany).

### 2.6 Fed‐Batch Characterization of KO Pools and Clones

To evaluate the changes in bioprocess performance of KO pools and clones, fed‐batch experiments were conducted by inoculating 25 mL 4Cell SmartCHO PM (Sartorius, Ulm, Germany) supplemented to 6 mM glutamine (Sartorius, Ulm, Germany) in 125 mL shake flasks (Corning, NY, USA) to 3E5 viable cells/mL. Cultures were fed with 4Cell SmartCHO FMA (Sartorius, Ulm, Germany) and 4Cell SmartCHO FMB (Sartorius, Ulm, Germany). The glucose concentration was adjusted to 5 g/L in addition to feeding. After 14 days or once viability reached below 70%, the culture was harvested to collect supernatant.

Antibody titer was quantified using the Octet HTX system (Sartorius, Göttingen, Germany). The antibody concentration of retention samples was calculated using a standard curve of the purified antibody.

### 2.7 Statistical Power Calculation

To determine the required sample size (*n*) for a given variation in process parameters, we calculated the sample size based on 90% power with a significance level of *α* = 0.05 for a specified difference in means, termed effect size (ES), using Equation (1). *Z* scores were calculated in Excel using the standard normal inverse function (NORM.S.INV) [27, 28].

Equation (1) Sample size calculation.

(1) $$n=\frac{2\sigma^{2}{\left(Z_{1-\frac{\alpha }{2}}+Z_{\beta }\right)}^{2}}{d_{\min}^{2}}$$

where *n* is the sample size, $Z_{1-\frac{\alpha }{2}}$ is the critical *Z* value corresponding to the desired significance level *α* (*α* = 0.05), *Z*_β_ is the *Z* value corresponding to desired statistical power *β* (*β* = 0.9), *d*_min_ is the minimum magnitude mean difference between groups (*d*_min_ = 0.3), σ is the standard deviation, α is the desired significance level *α* (*α* = 0.05), and β is the desired statistical power (*β* = 0.9).

## 3 Results and Discussion

### 3.1 Bypassing Single Cell Cloning for the Screening of KO Effects in Pools Saves 5–6 Weeks

**FIGURE 1:**
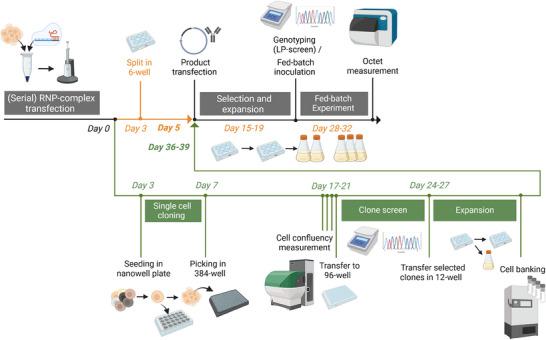
Workflow for the generation of host cell protein KO clones and subsequent performance capture in a fed‐batch bioprocess. (Created in BioRender. Marzluf, J. (2025) https://BioRender.com/s43e103). Further improvements in timeline and data integrity can be achieved by bypassing the single cell cloning directly to pooled fed‐batch evaluation. This reduces the workflow by 5–6 weeks. Furthermore, KO‐scores in bulk pools may be increased by employing sequential transfections with the same RNP‐complex. KO, knockout; RNP, ribonucleoprotein.

Traditional KO screening in CHO cells typically requires single‐cell cloning, a laborious process that requires multiple weeks of cultivation and screening [15, 16]. With the advancement of CRISPR tools, pooled approaches for screening have become more common. Here, a library of different sgRNA sequences is delivered into cells, generating a heterogenous pool which is then subjected to phenotypic or functional selection process. As a readout, the sgRNA count distribution is captured by NGS [29]. These pooled approaches have also been demonstrated in RNA interference screens [30]. Here, we aim to transfer the principle of using pools for screening purposes to our traditional KO platform process. As shown in Figure 1, this process includes multiple steps from seeding cells to clone expansion, which significantly delays phenotypic assessment. By directly screening KO effects in heterogeneous KO pools, the need for single cell cloning is eliminated, reducing the timeline to generate KO populations that can be used for downstream functional studies from 4 to 5 weeks to just 1 week.

To enable this, high KO efficiencies had to be achieved in pooled populations, and a linear relationship between genotype and phenotype as well as genotypic stability across the conducted process had to be shown. Such an approach not only accelerates the identification of KO phenotypes but also reduces labor and resource demands. It is particularly useful in multiplexed gene editing studies, allowing for efficient, early identification of impactful gene KO combinations.

### 3.2 Genotype and Phenotype Correlates in Heterogenous Fucosyltransferase 8 KO Pools

**FIGURE 2:**
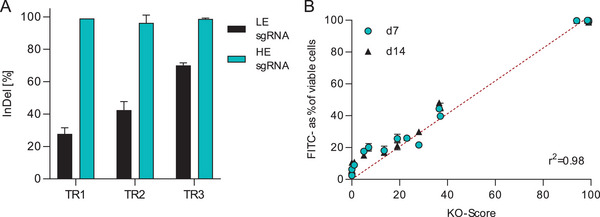
Multiple transfections increase KO‐efficiency and correlate with phenotypic loss of FUT8. (A) InDel percentages of FUT8 KO pools generated using LE and HE sgRNAs across one, two, and three sequential transfections (TR1–TR3), performed at 48‐h intervals. [*n* = 6; mean ± SD]. (B) Correlation between FUT8 KO‐scores and percentage of FITC‐LCA‐negative cells (FUT8‐deficient phenotype) measured at Days 7 and 14 after the final transfection. Each point represents a KO pool collected at the indicated time point after completing one, two, or three transfections. [*n* = 6; mean ± SD]. FITC‐LCA, fluorescein isothiocyanate labeled lens culinaris agglutinin; FUT8, α1,6‐fucosyltransferase 8; HE, high efficiency; KO, knockout; LE, low efficiency; SD, standard deviation; TR, transfection.

Achieving high KO efficiencies is crucial to effectively screen KO candidates for bioprocess behavior changes in heterogeneous KO pools. This is necessary to limit the masking effect of WT subpopulations present in KO pools. Sequential transfections were utilized to increase the efficiency of low‐ and high‐efficiency sgRNAs targeting the FUT8 gene, ultimately achieving significantly increased KO efficiencies after three sequential transfections. This method has been previously described in the context of a decaplex KO of CHO cell genes [16]. The FUT8 gene enables rapid identification of subpopulations not expressing functional FUT8 by analyzing the proportion of cells displaying nonfucosylated membrane proteins [10]. This commonly studied model gene catalyzes the addition of fucose to IgG1 antibodies in CHO cells. Removing FUT8 reduces antibody‐dependent cell‐mediated cytotoxicity [31, 32, 33]. Figure 2A shows the increase in InDel percentage for both a low efficiency (LE) and high efficiency (HE) sgRNA after one, two, and three sequential transfections. Gene editing transfections were carried out using the same RNP complex at 48‐h intervals. The HE sgRNA achieved a 99% ± 0% InDel frequency after a single transfection, while the LE sgRNA showed an increased from 28.0% ± 3.8% after the first transfection to 70.0% ± 1.6% InDel frequency following three transfections. The generated pools exhibited genotypic stability over time, with no detectable change in InDel profile across 2 weeks of cultivation (Figure 2B). The KO scores and phenotypic data shown in Figure 2B correspond to cell pools harvested at Days 7 and 14 following the final transfection of each sequential transfection series, respectively.

In the next step, we analyzed the connection between genotype and phenotype of KO pools. We used FUT8 again as a model gene, the LE and HE sgRNAs from the previous steps were applied to generate pools with varying KO scores. The KO score indicates the KO efficiency, representing the proportion of cells with either a frameshift InDel or a fragment deletion, which is likely to result in a functional KO. KO scores were determined using ICE analysis based on Sanger sequencing data. One and two weeks after transfection, the pools were stained with FITC‐LCA, the percentage of FITC‐negative cells was determined by FACS analysis and plotted against the measured KO scores. The KO scores correlated strongly with the percentage of FITC‐negative cells (Figure 2B), indicating that genotype modifications can reliably predict phenotypic outcomes (*r* ^2^ = 0.98). The described approach allows the identification of phenotypic effects resulting from KOs within heterogeneous CHO pools. However, for gene targets resulting in adverse effects on cell growth upon their KO, the KO population could be depleted from the cell pool, potentially resulting in false conclusions. Thus, monitoring KO scores throughout experimentation is important.

### 3.3 Heterogenous KO Pools Are Stable in Single and Multiplex Approaches

**FIGURE 3:**
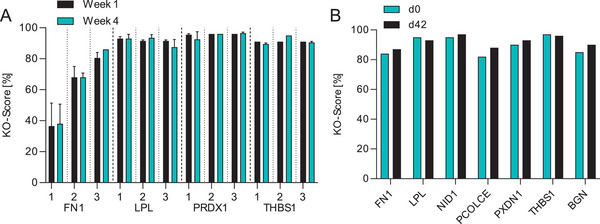
Stability of single and multiplexed KO pools over multiple weeks. (A) KO‐scores in single gene KO pools after one, two, and three sequential RNP‐complex transfections. Each subsequent column pair (black + blue bars) represents two pools after the respective number of transfections in the first week (black bars) and after four weeks (blue bars). [*n* = 2; mean ± SD]. (B) KO‐score in one 7x KO pool after three multiplexed transfections in the first week and after six weeks. [*n* = 1]. KO, knockout; RNP, ribonucleoprotein; SD, standard deviation.

To further investigate the stability of heterogeneous KO pools, four additional genes were evaluated in a singleplex approach. CHO cells underwent three sequential transfections, the pools were cultivated for 4 weeks and genotyped 1‐ and 4‐weeks posttransfection. Transfections with RNP complexes targeting the lipoprotein lipase (LPL), PRDX1, and thrombospondin 1 (THBS1) gene showed KO scores > 80% after a single transfection with additional transfections not further increasing the score. Transfected pools addressing the FN1 gene showed a marked increase from 36.5% ± 14.9% to 80.5% ± 3.5% after three transfections. KO scores for LPL, PRDX1, and THBS1 did not increase with subsequent transfections, likely due to the near‐complete editing of all WT sequences following the initial transfection. Cell viability remained above 90% throughout the sequential transfection process, with no detectable drop observed after any transfection round (data not shown), consistent with previous reports [16]. All pools show stability in KO scores, maintaining the initial KO score over a time period of at least 4 weeks (Figure 3A).

In a further experiment, we analyzed the stability of a 7x multiplex KO pool that was generated by transfecting mixtures of seven RNP complexes three sequential times, targeting the FN1, LPL, NID1, procollagen C‐endopeptidase enhancer (PCOLCE), peroxidasin 1 (PXDN1), THBS1, and BGN gene. This CHO multiplex KO pool was cultivated over 6 weeks. All KO scores remained above 80%, with some genes showing slight upward changes. However, these increases fell within the 5% method tolerance of ICE analysis following Sanger sequencing of cut site amplicons (Figure 3B) [34].

The generated CHO KO pools exhibited stability in both single and multiplexed approaches. The stability and linearity of genotype–phenotype correlations may vary depending on the target gene.

### 3.4 CHO KO Pools Are Better Suited Than Clones to Connect Genotype to Phenotype

**FIGURE 4:**
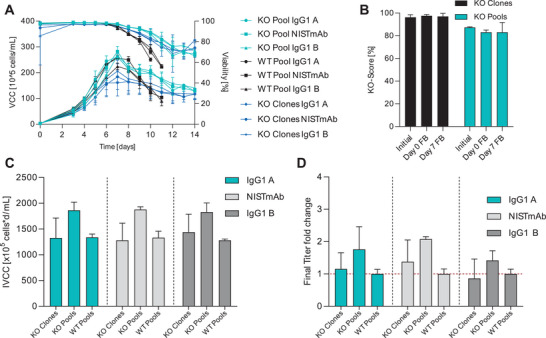
KO of FN1 increases final fed‐batch titer and prolongs the stationary phase in CHO clones and heterogenous KO pools. (A) Growth curves showing VCC and viability during shake‐flask fed‐batch with three different antibody coding genes. (*n* = 12 [FN1 KO clones], 3 [KO pools], 3 [WT pools]; mean ± SD). (B) KO scores along the cell line generation process until fed‐batch cultivation. (*n* = 12 [KO clones], [KO pools]; mean ± SD). (C) IVCC of all FN1 KO fed‐batch experiments. (*n* = 12 [FN1 KO clones], 3 [KO pools], 3 [WT pools]; mean ± SD). (D) Final titer fold change of all FN1 KO fed‐batch experiments. Titers were normalized against WT samples in their respective experiment and then plotted for the fold change observed. (*n* = 12 [FN1 KO clones], 3 [KO pools], 3 [WT pools]; mean ± SD). CHO, Chinese hamster ovary; FN1, fibronectin 1; IVCC, integral viable cell concentration; KO, knockout; SD, standard deviation; WT, wild‐type.

The FN1 gene, we previously identified as having bioprocess‐enhancing effects on cell phenotype (unpublished), was selected to compare the traditional single‐cell clone‐based approach with the pool‐based workflow (Figure 1). FN1 is part of the extracellular matrix and plays a key role in cell adhesion through interactions with integrins and downstream signaling [35]. In the absence of FN1, the integrin‐mediated adhesion signaling may be diminished and reduce the formation of cell aggregates through cell–cell interactions [36, 37]. In addition to cell adhesion, FN1 also regulates cell migration, proliferation, and survival by signaling through PI3K/Akt, MAPK/ERK signaling pathways [38]. Absence of FN1 via KO might contribute to an improved culture performance by delaying stress response that leads to increased cell aggregation and apoptosis. To validate our pool‐platform and reproduce these findings, we generated KO pools and single‐cell KO clones from the in‐house WT CHO DG44 cell line using an RNP complex targeting an early exon of the FN1 gene. In total, 96 single‐cell clones were generated for genotypic screening of which 12 clones were selected based on their KO genotype and clonal outgrowth. Clones were selected based on the presence of biallelic frameshift‐inducing InDels (i.e., insertions or deletions not divisible by three) in the target region, as determined by Sanger sequencing and ICE analysis, alongside robust clonal outgrowth. The FN1 KO pools were generated with the same RNP complexes that were used for KO clone generation with three sequential transfections to achieve high KO scores. To confirm that FN1 KO reduces protein expression, a Western blot was performed on KO pools generated using the same KO workflow. Relative FN1 levels, assessed by band intensities normalized to ACTB, showed an average 52% reduction in KO pools, supporting effective protein‐level disruption. Residual expression is consistent with KO scores below 100%, as some alleles may remain unedited or be heterozygous (Figure S1A, B). Both KO pools/clones and WT pools were used to generate stable production pools expressing three model IgG1 antibodies (NISTmAb, IgG1 A, and IgG1 B) through dihydrofolate reductase (DHFR) selection (National Institute of Standards and Technology [NIST] [39]). These production pools were then expanded for shake‐flask fed‐batch evaluations. Shake‐flask fed‐batch experiments were performed simultaneously with KO clones, KO pools, and WT pools for each of the three IgG1 molecules. During the fed‐batch bioprocess, both the KO clones and KO pools exhibited a delayed decline in viability, resulting in prolonged process duration and higher viability, which corresponds with an increased integral viable cell concentration (IVCC), ultimately also leading to higher titers (Figure 4A, C, D). The KO pools demonstrated similar growth dynamics to the WT up to the peak of viable cell concentration (VCC) on Day 7, followed by an extended stationary phase with high cell viability. This effect was also observed in the KO clones. However, the peak VCC varied considerably. The lower peak VCC can likely be attributed to clonal variation introduced during single‐cell isolation, as well as initial viability differences. For instance, in the case of IgG1 A, some KO clones exhibited starting viabilities as low as ∼80%, compared to FN1 KO pools and WT controls. Despite this variability, the KO clones maintained extended viability until the end of the process, with many replicates showing higher than 70% viability even on Day 14 of the fed‐batch run. The shift in process characteristics is thus consistently preserved in both KO clones and KO pools, demonstrating robust reproducibility across multiple IgG1 molecules and experiments. The productivity‐enhancing effect of FN1 KO was only partially reproduced in KO clones but was more evident in KO pools, which retain the full heterogeneity of the host population and are less prone to biases from random genetic or phenotypic variations that can arise during single‐cell cloning. The heterogeneous KO pools demonstrated a 1.4‐ to 2.1‐fold increase in final titer for the tested IgG1 molecules. Notably, this improvement was partially absent in KO clones, likely due to inherent cell background variation, which complicates the reproducibility of effects in individual clones. In contrast, KO pools capture the full heterogeneity of the host cell population, serving as a more accurate proxy for assessing changes across the entire host cell pool. By bypassing single‐cell cloning, which can introduce variability and noise, this approach offers substantial advantages for efficiently screening candidate KO genes. Additionally, the genetic stability of the KO pools was monitored. KO scores remained consistently high (>80%) for both KO clones and KO pools during the transfection, selection, and fed‐batch bioprocess (Figure 4B). This genetic stability through the KO screening process and fed‐batch evaluation is crucial for maintaining the desired phenotype for putative target discovery. These findings underscore the advantages of using KO pools for screening purposes. The heterogeneity of the pools appears to capture a wider range of phenotypes, potentially revealing improvements that might be missed when using individual clones. To efficiently utilize clones to make an accurate estimation of significant changes in titer would require the use of very high number of clones. Furthermore, the use of pools can reduce noise in the data and increase throughput in the screening process, as indicated in the challenges and improvements mentioned previously. The consistent productivity enhancement observed across different biotherapeutic proteins suggests that FN1 KO may have a broad applicability in improving CHO cell performance for various products.

### 3.5 Using Pools Instead of Clones for Genetic KO Screens Significantly Reduces Timelines and Increases Data Quality

**FIGURE 5:**
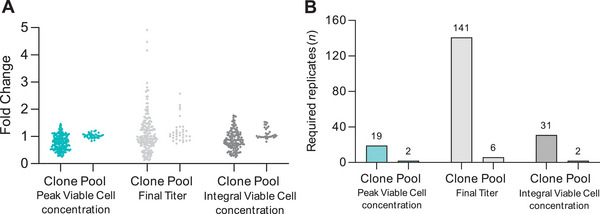
Empirical variance in fed‐batch bioprocess behavior comparing clones and pools. (A) Performance parameters peak viable cell concentration, final titer, and integral of viable cell concentration were normalized against WT samples in their respective experiment and then plotted for the fold change observed. (*n* = 175 [clone], 36 [pool]; mean ± SD). (B) Required number of replicates to resolve a 0.3‐fold mean difference between groups with 90% power in peak VCC, final titer, and IVCC. IVCC, increased integral viable cell concentration; SD, standard deviation; VCC, viable cell concentration; WT, wild‐type.

The use of KO pools in genetic screening studies offers significant advantages over traditional clonal approaches. When comparing empirical variability of clones and pools in our fed‐batch experiments across key bioprocess parameters: peak VCC, final titer, and IVCC, clones and pools behaved differently (Figure 5A). Clonal populations exhibited considerable heterogeneity across these key performance metrics, as evidenced by the wide range of data points for clones in all three parameters. Peak VCC in clonal populations varied from a 0.26‐ to 1.45‐fold change compared to the respective WT, while pooled populations exhibited a narrower range of 0.83–1.22. Similarly, final titers ranged from 0.12‐ to 4.91‐fold change in clones, while pools showed a more consistent range of 0.74–2.57. For IVCC, clones displayed a fold‐change range of 0.24–1.77, whereas pools demonstrated a more limited range from 0.81 to 1.53. The substantial variability observed in clonal populations complicates accurate assessment of genetic modifications, as selecting a limited number of clones may yield data that does not represent the overall population. In contrast, KO pools exhibit markedly reduced variability, with tighter clustering of data points—particularly in final titer—allowing for a more reliable view of the genetic modification's impact. This pooled approach captures a broader representation of the edited cell population, eliminating the bias and noise introduced by single‐clone selection. High variability in clonal data necessitates a large sample size to achieve adequate statistical power. Detecting a mean difference of 0.3 in final titer with 90% power would require 141 clones to be evaluated, while only six replicates would be sufficient in pooled populations to achieve the same result (Figure 5B). Although this discrepancy is less pronounced in peak VCC and IVCC, detecting significant effects still demands a 10–15 times higher replication in clones compared to pools. This requirement dramatically increases the resources, time, and effort needed for a reliable analysis. By using pools, fewer samples are required, yielding more reliable and representative data and thereby streamlining the evaluation process. Additionally, the pool‐based approach offers substantial time savings, reducing the timeline for assessing a single gene KO from 9 weeks with the clonal method to just 5 weeks. This efficiency also allows for a three‐fold increase in parallel gene screening capacity, enabling a faster and more comprehensive exploration of genetic targets. In summary, KO pools provide not only more consistent and representative data but also significantly enhance the efficiency of the cell line engineering process. This approach empowers us to make better‐informed decisions about genetic modifications, while greatly reducing the time and resources necessary for screening and evaluation.

The use of CHO pools has gained significant attention recently, driven by the urgent timeline demands imposed on biopharmaceutical development projects during the COVID‐19 pandemic. Several studies have demonstrated the feasibility and success of employing CHO production pools for clinical manufacturing, highlighting their potential to accelerate timelines without compromising product quality or yield [40, 41, 42]. These efforts underscore the robustness and scalability of CHO pools, validating their application for rapid, large‐scale production of clinical‐grade biologics and paving the way for broader adoption in accelerated drug development processes.

## 4 Conclusion

Genetically engineered CHO cell lines are poised to play a pivotal role in the future of biopharmaceutical production, offering enhanced process performance and superior product quality. Here, we have developed a streamlined and highly efficient pipeline for generating KO CHO cell lines with any desired gene KO. Our novel pooled screening approach has increased evaluation efficiency by six‐fold, enabling the rapid delivery of host cell lines with optimized productivity, growth characteristics, and product quality. These advancements pave the way for substantial improvements in process performance across the biopharmaceutical industry, with ongoing engineering efforts poised to further amplify these benefits. The pooled screening platform enables the efficient generation of CHO KO cell lines, facilitating detailed studies of production characteristics. Although the pooled KO approach offers a powerful tool for early stage functional screening and phenotype correlation, it is important to note that the final establishment of a production‐ready cell line still requires isolation and characterization of monoclonal populations. For example, if an FN1‐KO is identified as beneficial, a monoclonal FN1‐KO production cell line must be derived to ensure genetic homogeneity and stability for manufacturing. Thus, the pooled KO strategy serves as a rapid and scalable screening step within a broader cell line development workflow, ultimately supporting the generation of safer, more cost‐effective, and higher‐yielding CHO cell lines for industrial application.

## Author Contributions

**Jannis Peter Marzluf**: conceptualization, investigation, writing—original draft, methodology, validation, visualization, formal analysis, data curation, software. **Daniela Kirchmeier**: investigation, visualization, data curation, methodology. **Jennifer Klein**: investigation, visualization, formal analysis, methodology, data curation. **Christoph Zehe**: conceptualization, writing—review and editing, project administration, supervision, resources, funding acquisition. **Ann‐Cathrin Leroux**: conceptualization, writing—review and editing, project administration, supervision.

## Conflicts of Interest

The authors declare no conflicts of interest.

## Supporting information



Supporting information

## Data Availability

The datasets supporting the findings of this study are owned by Sartorius Stedim Cellca GmbH and contain proprietary information. Access may be granted to researchers upon reasonable request, subject to review and approval by Sartorius Stedim Cellca GmbH.
