# Supplementary material for: Utilizing Stable Gene‐Edited Knockout Pools for Genetic Screening and Engineering in Chinese Hamster Ovary Cells
Source: Biotechnol J. 2025 May 16;20(5):e70033. doi: 10.1002/biot.70033 (PMC12082383; doi:10.1002/biot.70033)
Supplement: Supplementary file 1 — Supporting information [file BIOT-20-e70033-s001.docx]

**Supplementary Information**
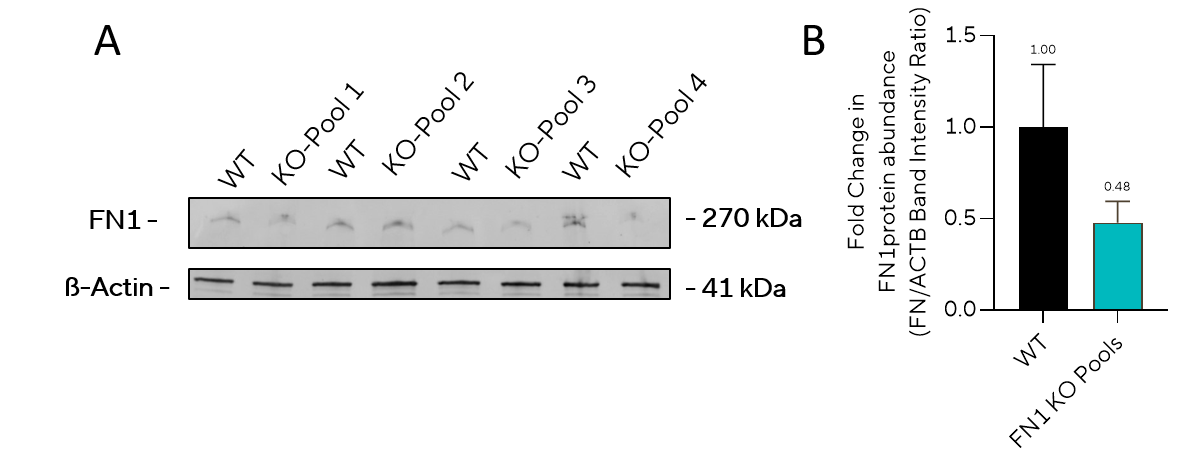


Supplementary Figure 1: **Confirmation of FN1 knockout at the protein level.** (A) Western blot showing FN1 protein abundance in WT and FN1 KO CHO cell pools. ACTB was used as a loading control. (B) Relative Change of FN1 band intensity normalized to ACTB, represented as fold change relative to WT (set to 1.0). Band Volume was quantified using GelAnalyzer 19.1. Data shows a mean 52% reduction in FN1 protein abundance in KO pools. Bars represent mean ± SD (n = 4 biological replicates). Western blot was performed using standard protocols. FN1 and ACTB were detected using commercially available antibodies (FN1: Sc-59826 AF680; ACTB: SC-47778 AF647); Lysates were prepared from ~100,000 cells per sample by direct boiling in 1× Laemmli buffer. 25,000 cell equivalents were loaded per lane.
